# Genome-Based Analysis of *Klebsiella* spp. Isolates from Animals and Food Products in Germany, 2013–2017

**DOI:** 10.3390/pathogens10050573

**Published:** 2021-05-08

**Authors:** Kathleen Klaper, Jens Andre Hammerl, Jörg Rau, Yvonne Pfeifer, Guido Werner

**Affiliations:** 1 Division Nosocomial Pathogens and Antibiotic Resistances, Department of Infectious Diseases, Robert Koch Institute, Wernigerode Branch, 38855 Wernigerode, Germany; pfeifery@rki.de (Y.P.); wernerg@rki.de (G.W.); 2 Unit Epidemiology, Zoonoses and Antimicrobial Resistance, Department Biological Safety, German Federal Institute for Risk Assessment (Bundesinstitut für Risikobewertung [BfR]), 12277 Berlin, Germany; Jens-Andre.Hammerl@bfr.bund.de; 3 Chemical and Veterinary Analysis Agency (CVUAS) Stuttgart, 70736 Fellbach, Germany; joerg.rau@cvuas.bwl.de

**Keywords:** *Klebsiella pneumoniae*, antibiotic resistance, virulence genes, aerobactin, pets, livestock, ESBL

## Abstract

The increase in infections with multidrug-resistant and virulent *Klebsiella pneumoniae* (*K. pneumoniae*) strains poses a serious threat to public health. However, environmental reservoirs and routes of transmission for *Klebsiella* spp. that cause infections in humans and in livestock animals are not well understood. In this study, we aimed to analyze the distribution of antibiotic resistance genes and important virulence determinants (*ybt*, *clb*, *iro*, *iuc*, *rmpA/A2*) among 94 *Klebsiella* spp. isolates from different animal and food sources isolated between 2013 and 2017 in Germany. Antibiotic susceptibility testing was performed, and the genomes were sequenced by Illumina and Nanopore technology. Genetic relationships were assessed by conducting core genome multilocus sequence typing (cgMLST). *Kleborate* was used to predict resistance and virulence genes; *Kaptive* was used to derive the capsule types. The results revealed that 72 isolates (76.6%) belonged to the *K. pneumoniae sensu lato* complex. Within this complex, 44 known sequence types (STs), 18 new STs, and 38 capsule types were identified. Extended-spectrum beta-lactamase (ESBL) genes were detected in 16 isolates (17.0%) and colistin resistance in one (1.1%) *K. pneumoniae* isolate. Virulence genes were found in 22 *K. pneumoniae* isolates. Overall, nine (9.6%) and 18 (19.1%) isolates possessed the genes *ybt* and *iuc*, respectively. Notably, aerobactin (*iuc* lineage 3) was only detected in *K. pneumoniae* isolates from domestic pigs and wild boars. This study provides a snapshot of the genetic diversity of *Klebsiella* spp. in animals and food products in Germany. The siderophore aerobactin was found to be more prevalent in *K. pneumoniae* strains isolated from pigs than other sources. Further investigations are needed to evaluate if pigs constitute a reservoir for *iuc* lineage 3.

## 1. Introduction

*Klebsiella* ssp. are Gram-negative, rod shaped, facultative anaerobic bacteria that belong to the *Enterobacteriaceae* family. They are ubiquitous in soil, surface waters, plants, and intestines of animals and humans [1,2]. The *Klebsiella pneumoniae* (*K. pneumoniae*) *sensu lato* complex is clinically the most problematic and comprises the phylogroups *K. pneumoniae* (Kp1), *K. quasipneumoniae* subsp. *quasipneumoniae* (Kp2), *K. quasipneumoniae* subsp. *similipneumoniae* (Kp4), *K. variicola* subsp. *variicola* (Kp3), *K. variicola* subsp. *tropica* (Kp5), *K. quasivariicola* (Kp6), and *K. africana* (Kp7) and [3,4]. *K. pneumoniae* is the most prevalent species isolated from human infections [4,5]. Classically, *K. pneumoniae* is considered to be an opportunistic pathogen, causing infections in immunocompromised patients, including urinary tract infections, pneumonia, and bloodstream infections (BSIs) [6,7]. *K. pneumoniae* is the second most common cause of BSIs caused by Gram-negative bacteria [8]. Due to the acquisition of multiple antibiotic resistance genes, the treatment of *K. pneumoniae* infections has become challenging and multidrug-resistant *Klebsiella* spp. are considered a public health threat—especially for predisposed persons [9,10,11]. In the last decades, *K. pneumoniae* has also been reported as a cause of community-acquired infections, including liver abscesses, endophthalmitis, and meningitis in otherwise healthy individuals [7,12]. These infections are often caused by hypervirulent isolates representing specific *K. pneumoniae* lineages, e.g., sequence types ST23 and ST66 [13,14]. Hypervirulent strains possess a distinct portfolio of virulence factors, mainly several siderophore genes, and express specific capsule types (e.g., K1 or K2) [4,15]. Furthermore, hypervirulent *K. pneumoniae* often demonstrate a hypermucoviscous phenotype that can be determined by a ‘string test’ on agar plates [16]. Distinct hypervirulent strains that have also acquired carbapenemase genes can enter hospitals and spread among patients. This has mainly been described for China but can also occasionally be observed in Central Europe, including in Germany [17].

In animals, *K. pneumoniae* can cause infections including pneumonia, mastitis, and bacteremia [18]. While the molecular and epidemiological characteristics of clinically important, multidrug-resistant *K. pneumoniae* STs in humans are well studied, little is known of the *K. pneumoniae* population structure in animals [4]. Livestock colonized by *K. pneumoniae* is thought to be a potential reservoir for antimicrobial resistance determinants and virulence factors and could facilitate the spread to human isolates [19]. The objective of this study was to investigate the molecular-genetic characteristics of *Klebsiella* spp. isolates from animal and food sources in Germany via whole-genome sequencing (WGS)-based analyses.

## 2. Results

### 2.1. Klebsiella spp. Population Analysis

**Figure 1:**
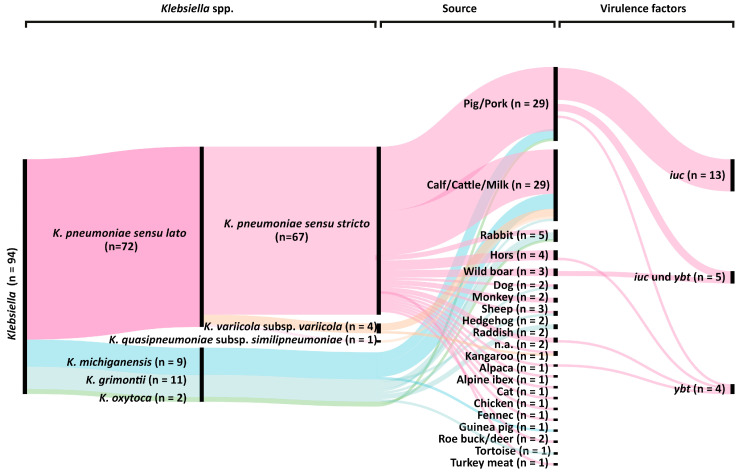
Genome-based classification of the 94 *Klebsiella* spp. isolates from animals and food. Graph includes species (1st column), phylogroups (2nd column; [3,4]), isolate sources (3rd column) and presence of specific virulence genes (4th column).

The study included 94 *Klebsiella* spp. isolates from animals and food sources of which most isolates (n = 58; 61.7%) were from pigs, pork, cattle, and milk. Furthermore, the collection contains isolates from vegetables, pets, livestock, and wild animals (Figure 1). MALDI-TOF biotyping suggested the presence of 69 *K. pneumoniae*, 21 *K. oxytoca*, and 4 *K. variicola*, using the commercial MALDI-biotyper database comprising 8468 entries. Subsequent WGS-based analysis revealed that one proposed *K. pneumoniae* and ten proposed *K. oxytoca* isolates were mis-assigned and instead belonged to the species *K. grimontii,* and nine proposed *K. oxytoca* isolates to the species *K. michiganensis.* All *K. grimontii* genomes contained the beta-lactamase gene *bla*_OXY 6_ which is known to be specific to this *Klebsiella* species [20,21]. Within the *K. pneumoniae sensu lato* complex, 67 (71.3%) isolates were identified as *K. pneumoniae*, four (4.3%) as *K. variicola* subsp. *variicola* and one (1.1%) as *K. quasipneumoniae* subsp. *similipneumoniae* (Figure 1).

### 2.2. Klebsiella spp. Antibiotic Resistance and Resistance Genes

**Figure 2:**
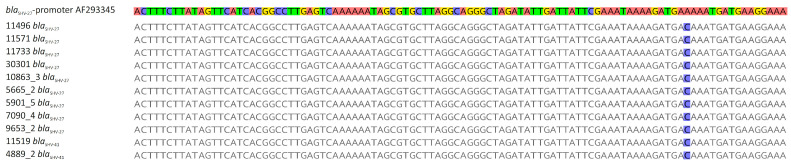
Comparison of the ‘wild type’ promoter of the ESBL gene *bla*_SHV-27_ (accession no. AF293345) and promoter sequences of *bla*_SHV-27/-41_ genes in eleven *K. pneumoniae* isolates of this study. These isolates were susceptible to third-generation cephalosporins (no ESBL phenotype).

The majority of the 94 study isolates (n = 73, 77.7%) were resistant to ampicillin. Resistance rates to third-generation cephalosporins, fluoroquinolones, and colistin were 5.3%, 3.2%, and 1.1%, respectively. Only one isolate showed resistance to all three classes (Supplementary Table S1). The genome-based prediction of resistance genes revealed the presence of various beta-lactamase genes (*bla*_SHV,_ *bla*_LAP_, *bla*_LEN_, *bla*_OKP-B_, *bla*_OXY_, *bla*_TEM_) in all but three *Klebsiella* spp. isolates (Table 1). Although only five isolates exhibited an ESBL phenotype (resistance to third-generation cephalosporins), WGS analyses identified 16 *K. pneumoniae* isolates that carried ESBL genes *bla*_SHV-27_ (n = 9), *bla*_SHV-41_ (n = 2), *bla*_CTX-M-1_ (n = 1), *bla*_CTX-M-14_ (n = 2), and *bla*_CTX-M-15_ (n = 2). However, subsequent sequence analysis of the *bla*_SHV-27_ and *bla*_SHV-41_ genes revealed a base-pair substitution (A to C) in all promoter sequences of these genes (Figure 2). The combination of the amino acid substitutions in GyrA (S83I) and ParC (S80I) (n = 2) and the acquired resistance genes *qnrB* (n = 1) and *qnrS* (n = 1) were detected in three isolates with resistance to the fluoroquinolone ciprofloxacin. Sequence analysis of the single colistin-resistant isolate showed that the chromosomal gene *mgrB* was interrupted by an IS*1*-family transposase gene. Resistance to carbapenems was not observed in the study isolates. 

### 2.3. Molecular Typing

**Figure 3:**
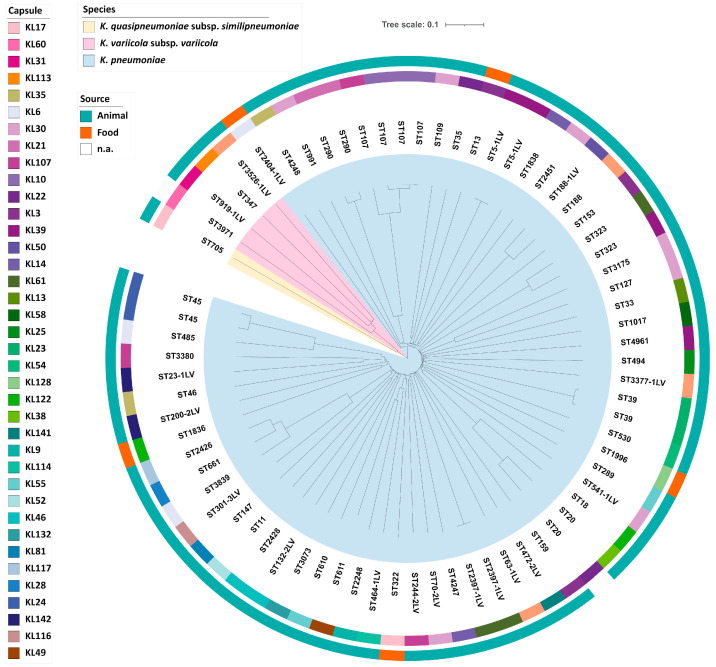
Phylogeny of 72 *K. pneumoniae sensu lato* isolates. The inner circle shows a Neighbor Joining tree based on cgMLST analysis. Each clade is colored according to the assigned species. Sequence types (STs) are labelled on the tree. The corresponding capsule locus (KL) and isolation sources are color-coded in the two outer rings (see legend).

MLST analysis of the 72 *K. pneumoniae sensu lato* complex isolates identified 62 STs, with ST107 (n = 4, 5.6%) as the most prevalent type (Table 2). Furthermore, 18 new STs were discovered. The genetic diversity was also reflected by capsule locus typing (Supplementary Table S1). The capsule loci KL30 (n = 7, 9.7%) was the most prevalentout of 38 capsule types (Figure 3). Furthermore, no association of specific ST or capsule type with isolation sources was identified. cgMLST revealed only three small clusters of genetically highly related isolates. Each cluster comprised only two isolates (Figure S1), which did not show more than eight allele differences. Overall, cgMLST analysis revealed no prevalent genetic lineage (Figure 3).

### 2.4. Presence of Virulence Factors

Genetic factors that are associated with hypervirulence were only detected in *K. pneumoniae* isolates. Overall, 22 *K. pneumoniae* genomes were positive for one or two siderophore systems (Figure 1). Yersiniabactin (*ybt*) was present in nine (9.6%) isolates and 18 isolates (19.1%) contained aerobactin (*iuc*) (Supplementary Table S1). Interestingly, aerobactin was only identified in domestic pig and wild boar isolates and belonged to the *iuc* lineage 3, whereas the presence of yersiniabactin was not associated with a specific isolation source. The addition of long-read sequencing for strain 30312,2 revealed a 171 kb IncFIB_K_ plasmid carrying *iuc*3. Subsequent sequence analysis showed that the IncFIB_K_ replicon was present in all *iuc*3 plasmids. Further virulence genes encoding for RmpA/A2, salmochelin, and colibactin were not detected in our study isolates.

## 3. Discussion

The analysis of 94 randomly collected *Klebsiella* spp. isolates from different animals and food sources showed that the species identification was not consistent between the two employed methods. Although MALDI-TOF spectrometry is commonly used in veterinary and medical microbiology laboratories, its discriminatory power depends on the underlying database, which requires continuous curation and updates, especially when closely related taxa such as *Klebsiella* spp. are analyzed. In this case, WGS-based analyses are better suited to species identification [4,5,22]. In accordance with previous studies, a substantial proportion of the study isolates (n = 67, 71.3%) were classified as *K. pneumoniae sensu stricto*, suggesting a wide prevalence also in a veterinary context. Such isolates appeared as food colonizers or as infectious agents among livestock animals [23,24,25]. 

*Klebsiella* spp. are rarely detected in animals and food samples [4]. Nevertheless, there are reports of sequence types ST11, ST15, ST25, and ST23 being isolated from livestock and companion animals [26,27,28,29,30,31]. These globally distributed STs are associated with the majority of nosocomial and community-acquired *K. pneumoniae* infections in humans [4]. In this study, we detected one *K. pneumoniae* ST11 and one ST147 isolate in pig and cattle, respectively. Since these STs are proposed to be human-associated, it is conceivable that they were transmitted from humans to animals [32]. Further human-associated or hypervirulent STs were not detected. However, our study collection contains a high genetic diversity, with 62 STs among 67 *K. pneumoniae sensu stricto* isolates. From this set, no predominant host-associated sequence type or reservoir could be determined. ST107 (four isolates, three with capsule type KL10) was the most prevalent ST among the 94 study isolates. *K. pneumoniae* of this ST have been occasionally isolated from human patients [33,34,35]. 

Antibiotic use in veterinary medicine is thought to increase the risk of antibiotic-resistant bacteria that can be transmitted to humans [36]. High prevalence rates of *Escherichia coli* with ESBL-mediated resistance to third-generation cephalosporins and MCR-1-mediated colistin resistance have been reported from livestock and food in recent years [37,38,39]. As expected, the resistance to ampicillin was frequent among the study collection due to the presence of intrinsic beta-lactamase genes in *Klebsiella* spp. (*bla*_SHV_, *bla*_OXY_, *bla*_OKP_, *bla*_LEN_). ESBL genes were detected in 16 of 94 isolates, but only five isolates with *bla*_CTX-M_ genes showed resistance to third-generation cephalosporins (ST11, ST13, ST107, ST1017, ST1836). The remaining eleven isolates harbored *bla*_SHV-27_ and *bla*_SHV-41_ genes, but also a base-pair substitution in their promoter sequence, that has been described to be associated with a weak *bla* gene expression and non-ESBL phenotype [40]. Resistance to colistin was detected in only one (ST11, pig) of the 94 *Klebsiella* spp. isolates, but the underlying mechanism was not MCR-1-mediated, as described for *E. coli* from livestock [39]. Instead, an insertion in the *mgrB* gene was detected, which results in a truncated and most likely non-functional protein [41]. Changes in the *mgrB* gene have frequently been described as a cause of colistin resistance in *K. pneumoniae* [42].

Virulence genes were present in only 22 *K. pneumoniae* isolates. Interestingly, the *iuc* gene encoding the siderophore aerobactin was associated with *K. pneumoniae* isolates of pig and wild boar origin (Figure 1, Table S1). Although these isolates differed in ST and capsule type, all harbored the *iuc3* gene. The *iuc* lineage 3 is associated with self-transmissible IncFIB_K_ and IncFII_K_ virulence plasmids [43]. Since all 22 *iuc3* isolates harbored plasmids of the IncFIB_K_ type, we believe that domestic pigs could be a reservoir for *K. pneumoniae* carrying *iuc3* on plasmids. However, this hypothesis requires further experimental proof using larger study collections.

## 4. Materials and Methods

### 4.1. Bacterial Isolates

A total of 90 *Klebsiella* spp. isolates were collected by the Chemical and Veterinary Analysis Agency Stuttgart between 2013 and 2017 as part of their routine diagnostic activities, one isolate was added from the Bundesinstitut für Risikobewertung (BfR) and three additional isolates from 2004 and 2006, respectively, were included as reference isolates. Further information about the isolates can be found on MALDI-UP [44].

### 4.2. Species Identification and Antibiotic Susceptibility Testing (AST)

*Klebsiella* species identification was conducted using a Bruker Microflex LT MALDI TOF MS after performing an extraction protocol in combination with the Biotyper database “MBT 8,468 MSP library” as recommended by the manufacturer. The species was assigned if the first two hits matched and showed a score >2.0. AST was performed using the broth microdilution assay according to CLSI guidelines. An interpretation of the minimal inhibitory concentrations was carried out using the European Committee on Antimicrobial Susceptibility Testing (EUCLAST) clinical breakpoints v10 derived for the human clinical context (http://www.eucast.org/clinical_breakpoints, accessed on 25 March 2021) (Supplementary Table S1). 

### 4.3. Whole Genome Sequencing (WGS) and De Novo Assembly

Bacteria were cultivated at 37 °C for 16–20 h under shaking conditions in lysogeny broth (LB). Genomic DNA was extracted from overnight cultures using the Purelink Genomic DNA Mini Kit (Thermo Fisher Scientific Inc., Waltham, MA, USA), in line with the manufacturer’s instructions. Quality evaluation and DNA quantification was carried out with the Nanodrop 1000 and the Qubit 4.0 system (Thermo Fisher), respectively. Sequencing libraries were prepared using the Nextera XT DNA Flex Sample Preparation Kit and sequenced using the MiSeq reagent v3 600 cycle kit for paired-end sequence determination (2 × 300 bp) according to the manufacturer’s protocol, on a Miseq benchtop device (Illumina Inc., San Diego, CA, USA). Read qualities were assessed using the pipeline *QCumber* (v2.1.1) where the *FastQC* (v0.11.5) and *Kraken* (v0.10.6) tools are implemented [45]. Raw reads were trimmed using *Trimmomatic* (v0.36; options ‘sliding window 4:20′) [46] and de novo assembled with *SPAdes* (v3.12.0) [47]. In addition, an *iuc3*-positive isolate was subjected to long-read sequencing by MinION (Oxford Nanopore Technologies, Oxford, UK). A hybrid assembly of Illumina and MinION sequence data was done using Unicycler (v0.4.4; https://github.com/rrwick/Unicycler, accessed on 25 March 2021) [48]. The plasmid sequence was annotated by using the Geneious Prime software (v2020.2.3; Biomatters Ltd., Auckland, New Zealand).

### 4.4. WGS-Based Typing and Virulence and Resistance Gene Prediction

For phylogenetic analysis, the *Ridom* software *SeqSphere+* v7.1.0 (Ridom GmbH, Münster, Germany) was used. The assembled genomes were uploaded to for multilocus sequence typing (MLST). New sequence types were assigned through *SeqSphere*+. The assignment to cluster types was done using a *SeqSphere+* defined *K. pneumoniae sensu lato* core genome MLST (cgMLST) scheme from which a neighbor-joining phylogenetic tree was generated. The resulting tree was visualized with the *iTOL* software (v5.6.1) [49]. Identification of capsule loci was done using *Kaptive* (http://kaptive.holtlab.net/, accessed on 25 March 2021). The presence of known virulence factors (*clb, ybt, iro, iuc, rmpA/rmpA2*) and various resistance genes was determined with the *Kleborate* tool (https://github.com/katholt/Kleborate, accessed on 25 March 2021) and *SeqSphere+*.

## 5. Conclusions

This study demonstrates the genetic diversity of *Klebsiella* spp. isolates from animals and food products. Despite this diversity, we identified several well-known epidemic clones associated with nosocomial infections and multidrug resistance in humans, such as *K. pneumoniae* ST11 and ST147. The investigation of antibiotic resistance genes showed that the presence of the ESBL genes *bla*_SHV-27_ and *bla*_SHV-41_ did not result in resistance to third generation cephalosporins due to a mutation in their promoter sequences. The virulence factor aerobactin was the most prevalent siderophore locus in our collection, and our data suggest that pig isolates may act as a reservoir for the siderophore *iuc* lineage 3 in *K. pneumoniae*.

## Figures and Tables

**Table 1:** Distribution and prevalence of different beta-lactamase genes, including ESBL genes among the 94 *Klebsiella* spp. isolates from animals and food.

Beta-Lactam Resistance	Isolates
	n = 94
**Beta-lactamase genes**	
*bla*_SHV-1_	9 (9.6%)
*bla*_SHV-11_	27 (28.7%)
*bla*_SHV-33_	1 (1.1%)
*bla*_SHV-37_	2 (2.1%)
*bla*_SHV-61_	1 (1.1%)
*bla*_SHV-108_	2 (2.1%)
*bla*_SHV-119_	1 (1.1%)
*bla*_SHV-157_	1 (1.1%)
*bla*_SHV-168_	1 (1.1%)
*bla*_SHV-187_	1 (1.1%)
*bla*_SHV-194_	1 (1.1%)
*bla*_SHV-199_	2 (2.1%)
*bla*_SHV-211_	1 (1.1%)
*bla*_SHV-215_	1 (1.1%)
*bla*_SHV-234_	1 (1.1%)
*bla*_SHV-244_	1 (1.1%)
*bla*_LAP-2_	2 (1.1%)
*bla*_LEN-16_	1 (1.1%)
*bla*_LEN-19_	1 (1.1%)
*bla*_LEN-24_	1 (1.1%)
*bla*_LEN-32_	1 (1.1%)
*bla*_OKP-B-6_	1 (1.1%)
*bla*_OXA-1_	2 (2.1%)
*bla*_OXY1-1_	3 (3.2%)
*bla*_OXY1-4_	3 (3.2%)
*bla*_OXY1-5_	2 (2.1%)
*bla*_OXY2-1_	2 (2.1%)
*bla*_OXY5-1_	1 (1.1%)
*bla*_OXY6-2_	4 (4.3%)
*bla*_OXY6-3_	1 (1.1%)
*bla*_OXY6-4_	6 (6.4%)
*bla*_TEM-1_	3 (3.2%)
**Extended-spectrum beta-lactamase genes**	
*bla*_SHV-27_ *	9 (9.6%)
*bla*_SHV-41_ *	2 (2.1%)
*bla*_CTX-M-1_	1 (1.1%)
*bla*_CTX-M-14_	2 (2.1%)
*bla*_CTX-M-15_	2 (2.1%)

* These isolates did not exhibit the ESBL phenotype with resistance to third-generation cephalosporins due to a base-pair substitution (A to C) in the promoter sequences of these genes (Figure 2).

**Table 2:** Diversity of multilocus sequence types among the 72 *K. pneumoniae sensu lato* isolates.

Sequence Types		Novel Sequence Types	
ST	n = 72	ST	n = 72
ST11	1 (1.4%)	ST5-1LV	2 (2.8%)
ST13	1 (1.4%)	ST23-1LV	1 (1.4%)
ST18	1 (1.4%)	ST63-1LV	1 (1.4%)
ST20	2 (2.8%)	ST70-2LV	1 (1.4%)
ST33	1 (1.4%)	ST132-2LV	1 (1.4%)
ST35	1 (1.4%)	ST188-1LV	1 (1.4%)
ST39	2 (2.8%)	ST200-2LV	1 (1.4%)
ST45	2 (2.8%)	ST244-2LV	1 (1.4%)
ST46	1 (1.4%)	ST301-3LV	1 (1.4%)
ST107	4 (5.6%)	ST464-1LV	1 (1.4%)
ST109	1 (1.4%)	ST472-2LV	1 (1.4%)
ST127	1 (1.4%)	ST541-1LV	1 (1.4%)
ST147	1 (1.4%)	ST919-1LV	1 (1.4%)
ST153	1 (1.4%)	ST2397-1LV	2 (2.8%)
ST159	1 (1.4%)	ST2404-1LV	1 (1.4%)
ST188	1 (1.4%)	ST3377-1LV	1 (1.4%)
ST289	1 (1.4%)	ST3381-1LV	1 (1.4%)
ST290	2 (2.8%)	ST3526-1LV	1 (1.4%)
ST322	1 (1.4%)		
ST323	2 (2.8%)		
ST347	1 (1.4%)		
ST485	1 (1.4%)		
ST494	1 (1.4%)		
ST530	1 (1.4%)		
ST610	1 (1.4%)		
ST611	1 (1.4%)		
ST661	1 (1.4%)		
ST705	1 (1.4%)		
ST991	1 (1.4%)		
ST1017	1 (1.4%)		
ST1836	1 (1.4%)		
ST1838	1 (1.4%)		
ST1996	1 (1.4%)		
ST2248	1 (1.4%)		
ST2426	1 (1.4%)		
ST2428	1 (1.4%)		
ST2451	1 (1.4%)		
ST3073	1 (1.4%)		
ST3175	1 (1.4%)		
ST3380	1 (1.4%)		
ST3839	1 (1.4%)		
ST3971	1 (1.4%)		
ST4247	1 (1.4%)		
ST4248	1 (1.4%)		

nLV (locus variant), number of loci this strain differs from the closest reported sequence type (ST) (https://github.com/katholt/Kleborate/wiki/MLST; v. 0.4.0-beta, accessed on 25 March 2021).

## Data Availability

Information about the isolates used and the MALDI-TOF MS single spectra are listed on the MALDI-UP website https://maldi-up.ua-bw.de/, accessed on 25 March 2021 [44]. The nucleotide sequences of the *Klebsiella* spp. isolates of this study are deposited in GenBank. The accession numbers are specified in Supplementary Table S1.

## Supplementary Materials

The following table is available online at https://www.mdpi.com/2076-0817/10/5/573/s1, Table S1: Characteristics of 94 *K. pneumoniae* isolates from different animals and food products, *2013*–*2017*, Germany.
